# Impact of in vitro digested zinc oxide nanoparticles on intestinal model systems

**DOI:** 10.1186/s12989-022-00479-6

**Published:** 2022-05-30

**Authors:** Anna Mittag, Alina Singer, Christian Hoera, Martin Westermann, Alexander Kämpfe, Michael Glei

**Affiliations:** 1 Department of Applied Nutritional Toxicology, Institute of Nutritional Sciences, Friedrich Schiller University, Jena, Germany; 2 Swimming and Bathing Pool Water, Chemical Analytics, German Environment Agency, Bad Elster, Germany; 3 Electron Microscopy Center, University Hospital, Friedrich Schiller University, Jena, Germany

**Keywords:** Caco-2, Coculture, HT29-MTX, In vitro digestion, Nanoparticles, Toxicity, Transwell, Zinc oxide

## Abstract

**Background:**

Zinc oxide nanoparticles (ZnO NP) offer beneficial properties for many applications, especially in the food sector. Consequently, as part of the human food chain, they are taken up orally. The toxicological evaluation of orally ingested ZnO NP is still controversial. In addition, their physicochemical properties can change during digestion, which leads to an altered biological behaviour. Therefore, the aim of our study was to investigate the fate of two different sized ZnO NP (< 50 nm and < 100 nm) during *in vitro* digestion and their effects on model systems of the intestinal barrier. Differentiated Caco-2 cells were used in mono- and coculture with mucus-producing HT29-MTX cells. The cellular uptake, the impact on the monolayer barrier integrity and cytotoxic effects were investigated after 24 h exposure to 123–614 µM ZnO NP.

**Results:**

In vitro digested ZnO NP went through a morphological and chemical transformation with about 70% free zinc ions after the intestinal phase. The cellular zinc content increased dose-dependently up to threefold in the monoculture and fourfold in the coculture after treatment with digested ZnO NP. This led to reactive oxygen species but showed no impact on cellular organelles, the metabolic activity, and the mitochondrial membrane potential. Only very small amounts of zinc (< 0.7%) reached the basolateral area, which is due to the unmodified transepithelial electrical resistance, permeability, and cytoskeletal morphology.

**Conclusions:**

Our results reveal that digested and, therefore, modified ZnO NP interact with cells of an intact intestinal barrier. But this is not associated with serious cell damage.

## Background

Due to their advantageous properties, enormous amounts of nanoparticles (NP) are produced and extensively applied each year, which leads to a steady rise in the discharge to the ecological system [1]. In particular, the food sector offers comprehensive application areas for NP, for instance in food packaging materials, as nano-pesticides, and other food-related products [2]. Therefore, there is an increasing amount of NP in the human food chain, and oral ingestion reflects one of the main routes of human exposure to NP [3].

Zinc oxide (ZnO) NP have some beneficial characteristics, such as high thermal, chemical and mechanical stability as well as antimicrobial and photocatalytic activity [4–6]. Therefore, they are used in consumer-related areas, for example in food storage containers, for food processing or food preservation, as dietary supplements instead of zinc salts, and as food additives. They can also be added to milk, dairy products, cereals, and beverages [2, 7, 8]. The widespread production and application of ZnO NP unavoidably lead to the release of these particles into the environment [9].

During the last few years, the environmental behaviour and bio-toxicity of NP and especially ZnO NP have gained increasing attention [1, 7, 9]. Meanwhile, there are some concerns about the potential toxicity induced by NP used in the food sector [2]. Thus, there is an urgent need for knowledge of NP interactions with biological systems to find key parameters for the prediction of NP reactivity and, thus, nanotoxicity [10].

Previously published data on toxic effects of orally ingested ZnO NP on intestinal models are still heterogeneous. Some studies identified negative biological consequences, for example an increased intestinal inflammation, decreased cell viability and depolarization of mitochondrial membranes, induced by the ZnO NP treatment [11, 12]. Others showed rather inconclusive results that could not demonstrate ZnO NP toxicity in some assays, for instance an unmodified monolayer permeability but a disrupted brush border membrane [13–17], or concluded the innocuousness of a ZnO NP exposure [18–23]. The toxicity seems to depend on the used cell model, among other factors [24].

It is already known that the physicochemical properties of NP can change when they are used as food additives [2], and especially after oral intake and digestion, the NP characteristics and also their behaviour, agglomeration, deagglomeration and dissolution capabilities, and consequently, their toxicological potential for human and ecological health can be affected due to the physiological environment of the human digestive system, which is characterized by changing pH values and diverse mixtures of salts and enzymes [3, 10]. The composition of the ingested food should also be considered as it can also influence the NP properties during digestion [3]. Logically, to assess the consequences of intestinal uptake on the properties and potential toxicity of ZnO NP, imitating the digestion process is useful. This is necessary since the properties of undigested and digested ZnO NP can differ and thus also their influence on intestinal cells [3, 8]. Available data about the influence of the digestion process on physicochemical properties and the biological behaviour of ZnO NP are insufficient [3]. But the comprehension of the fate of NP after ingestion is relevant to contextualize the effects of NP in the gastrointestinal tract [25, 26].

Therefore, two different sized ZnO NP were *in vitro* digested to approximate physiological circumstances. The digested ZnO NP were characterized and taken for cell culture experiments in relevant concentrations. For this, differentiated Caco-2 cells, which are morphologically and functionally similar to enterocytes *in vivo*, were used as monoculture. In addition, the mucus-producing goblet cells HT29-MTX were added as coculture to resemble the complex structure of the gastrointestinal tract more closely. The cells were grown on Transwell inserts, which allow separation into apical and basolateral compartments. After treatment of the cells with the digested ZnO NP, zinc uptake and effects on barrier integrity, permeability, metabolic activity, generation of reactive oxygen species (ROS), and the mitochondrial membrane potential (MMP) were investigated.

## Results

### Characterization of ZnO NP

**Fig. 1:**
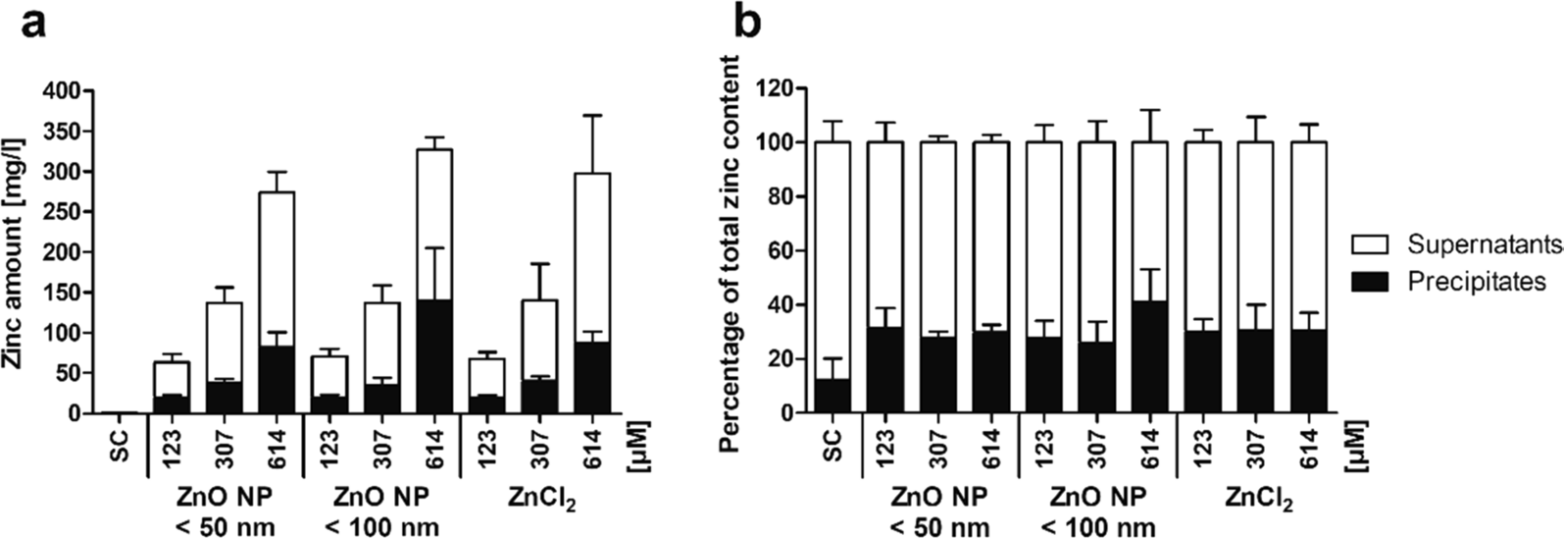
Proportion of free zinc ions (supernatants; white bars) and bound zinc (precipitates; black bars) in the intestinal juice after *in vitro* digestion of zinc oxide nanoparticles (ZnO NP < 50 and < 100 nm) or zinc chloride compared to the solvent control (= SC, intestinal juice), represented as total amount of zinc (**a**) and percentage of the total zinc content (**b**). Data are expressed as mean + SD, n = 4

The proportion of free zinc ions and bonded zinc in the artificial intestinal juices were investigated after simulating the mouth, gastric and intestinal phase using ICP-OES. The total amount of zinc in the pure intestinal juice (Fig. 1a) was 0.8 ± 0.1 mg/l in the supernatants and 0.1 ± 0.1 mg/l in the precipitates. The amount of zinc in the intestinal juice increased with increasing applied ZnO NP and ZnCl_2_ quantities. After in vitro digestion of 614 µM ZnO NP and ZnCl_2_, the amount of zinc was more than 200-fold higher in the supernatants (white bars; 192.0 ± 25.2 mg/l for ZnO NP < 50 nm, 187.5 ± 15.8 mg/l for ZnO NP < 100 nm, 209.7 ± 72.2 mg/l for ZnCl_2_) and more than 600-fold higher in the precipitates (black bars; 82.3 ± 18.7 mg/l for ZnO NP < 50 nm, 139.6 ± 65.6 mg/l for ZnO NP < 100 nm, 87.4 ± 14.0 mg/l for ZnCl_2_) compared with the pure intestinal juice. About 70% of the total amount of zinc was found in the supernatants and only 30% of the zinc was detectable in the precipitates (Fig. 1b). There were only minor differences between ZnO NP and ZnCl_2_ regarding the proportions of free zinc ions and bound zinc.

**Fig. 2:**
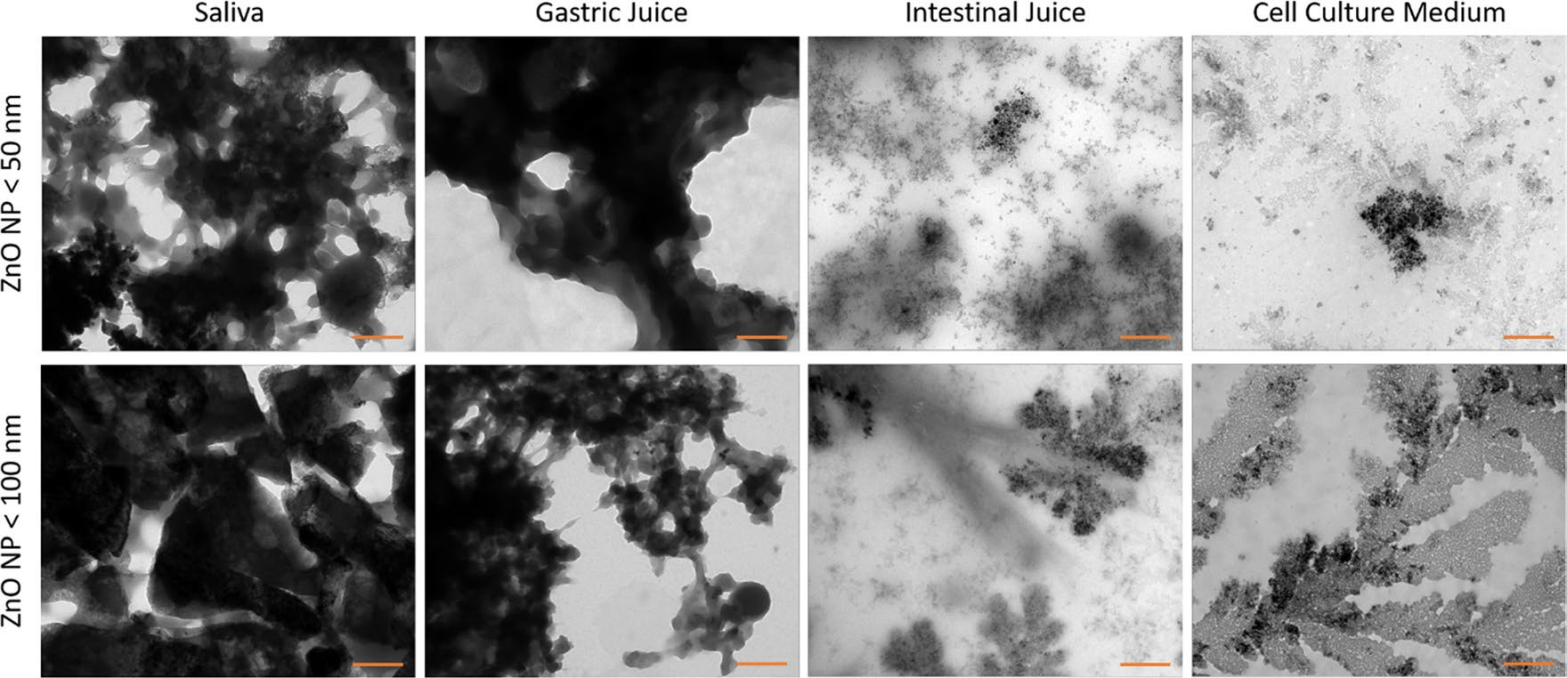
Representative transmission electron microscopy (TEM) images of ZnO NP < 50 nm (upper row) and < 100 nm (lower row) during and after in vitro digestion. Samples were taken after the mouth, gastric, and intestinal phase and after 1:10 dilution of the intestinal juice with the cell culture medium. Orange scale bar: 250 nm

TEM was used to investigate and visualize the fate of ZnO NP (< 50 nm and < 100 nm) during the *in vitro* digestion steps (Fig. 2). In the saliva and gastric juice, there were complex structures with a high contrast because of the high ZnO NP concentration (3.34 mg/ml). The aggregates of ZnO NP < 50 nm differed morphologically from that of ZnO NP < 100 nm. After the intestinal phase and the final dilution with the cell culture medium, structural changes occurred. There was an obvious structural shift of the NP from the mouth phase (saliva) until dilution with the cell culture medium.

### Quantification of zinc

**Fig. 3:**
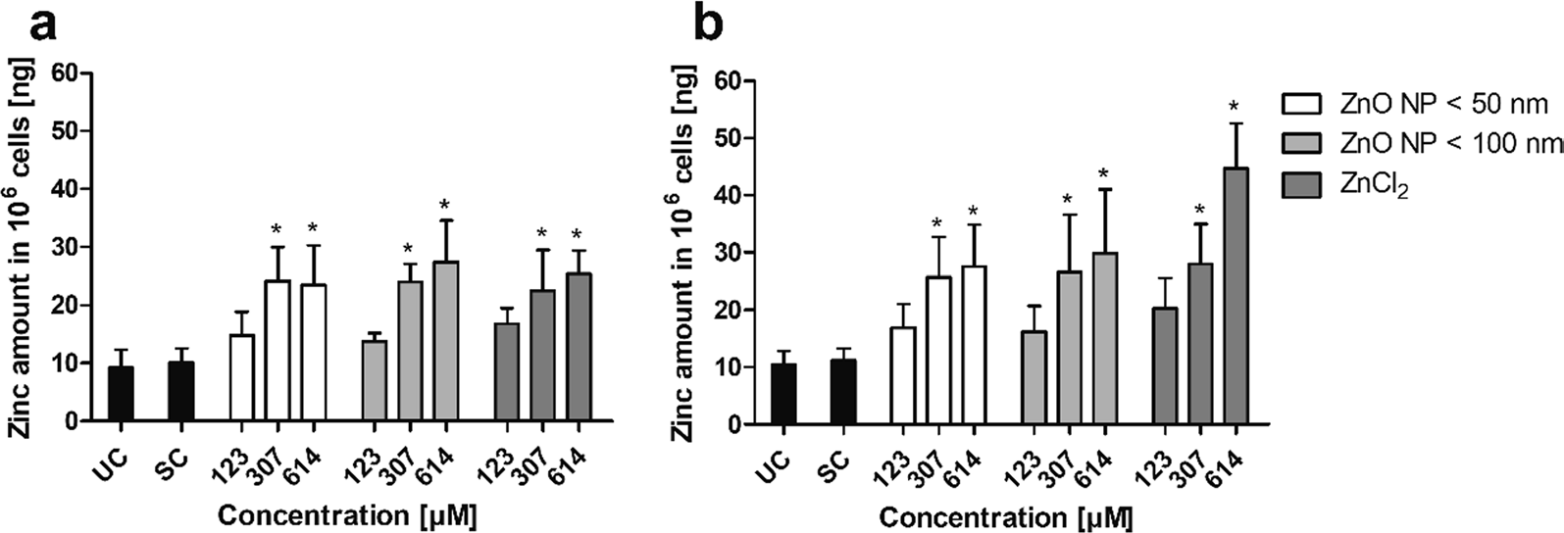
Amount of zinc in Caco-2 monocultured (**a**) and Caco-2/HT29-MTX cocultured cells (**b**) after 24 h treatment with digested ZnO NP (< 50 nm and < 100 nm) and ZnCl_2_. UC: untreated control; SC: solvent control (10% intestinal juice). Data are expressed as mean + SD; n = 5. Significant differences compared to SC (* P ≤ 0.05) were obtained by one-way analysis of variance/Ryan–Einot–Gabriel–Welsh post hoc test

The cellular uptake of digested ZnO NP was investigated using ICP-MS (Fig. 3). Therefore, the cellular amount of zinc was calculated for 1 × 10^6^ cells. The untreated and solvent control showed similar zinc quantities in both cultures (monoculture: 9.2 ± 3.1 ng zinc in the untreated control and 10.1 ± 2.5 ng zinc in the solvent control; coculture: 10.5 ± 2.3 ng zinc in the untreated control and 11.2 ± 2.1 ng zinc in the solvent control). There was a significant increase in the amount of zinc in both cultures after treatment with 307 µM and 614 µM ZnO NP < 50 nm, ZnO NP < 100 nm, and ZnCl_2_. In the monoculture (Fig. 3a), incubation with 614 µM ZnO NP < 100 nm led to a 2.7-fold and, thereby, highest increase (27.4 ± 7.1 ng zinc). In the coculture (Fig. 3b), there was a fourfold and, thereby, highest increase after treatment with 614 µM ZnCl_2_ (44.7 ± 7.9 ng zinc).

**Fig. 4:**
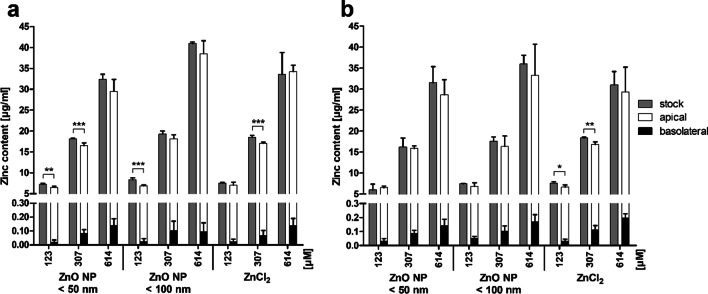
Zinc content of apical and basolateral supernatants of Caco-2 monocultured (**a**) and Caco-2/HT29-MTX cocultured cells (**b**) after 24 h of treatment with digested ZnO NP (< 50 nm and < 100 nm) and ZnCl_2_ compared to the stock dispersions. Data are expressed as mean + SD; n = 5. Significant differences between the amounts of zinc before (stock) and after incubation (apical + basolateral; *P ≤ 0.05; **P ≤ 0.01; ***P ≤ 0.001) were obtained by using the unpaired t test

Zinc permeation through the monolayer of mono- and cocultured cells after treatment with digested ZnO NP was investigated using ICP-MS (Fig. 4). Therefore, the zinc content of the incubation medium was measured before and after treatment of the cells from the apical and basolateral compartments. Data were corrected for the basal zinc content of the intestinal juice solvent control (1:10 diluted with cell culture medium). The zinc content of the incubation medium increased with increasing applied ZnO NP and ZnCl_2_ quantities. There were only small amounts of zinc in the basolateral compartments with the highest amounts after treatment with 614 µM ZnO NP and ZnCl_2_ (0.1 ± 0.0 µg/ml zinc in the monoculture; 0.2 ± 0.0 µg/ml zinc in the coculture). The differences between the stock dispersion and the sum of apical and basolateral dispersions reflect the cellular zinc uptake. In the monoculture (Fig. 4a), there were significant differences detectable after treatment with 123 µM ZnO NP < 50 nm (stock: 7.3 ± 0.3 µg/ml zinc; apical: 6.5 ± 0.3 µg/ml zinc; basolateral: 0.03 ± 0.0 µg/ml zinc), 307 µM ZnO NP < 50 nm (stock: 18.2 ± 0.2 µg/ml zinc; apical: 16.5 ± 0.6 µg/ml zinc; basolateral: 0.1 ± 0.0 µg/ml zinc), 123 µM ZnO NP < 100 nm (stock: 8.4 ± 0.4 µg/ml zinc; apical: 6.9 ± 0.2 µg/ml zinc; basolateral: 0.02 ± 0.0 µg/ml zinc), and 307 µM ZnCl_2_ (stock: 18.5 ± 0.4 µg/ml zinc; apical: 17.0 ± 0.2 µg/ml zinc; basolateral: 0.1 ± 0.0 µg/ml zinc). The cocultured cells (Fig. 4b) showed significant differences after treatment with 123 µM ZnCl_2_ (stock: 7.6 ± 0.3 µg/ml zinc; apical: 6.7 ± 0.5 µg/ml zinc; basolateral: 0.03 ± 0.0 µg/ml zinc) and 307 µM ZnCl_2_ (stock: 18.4 ± 0.3 µg/ml zinc; apical: 16.7 ± 0.7 µg/ml zinc; basolateral: 0.1 ± 0.0 µg/ml zinc).

### Transmission electron microscopy evaluation

**Fig. 5:**
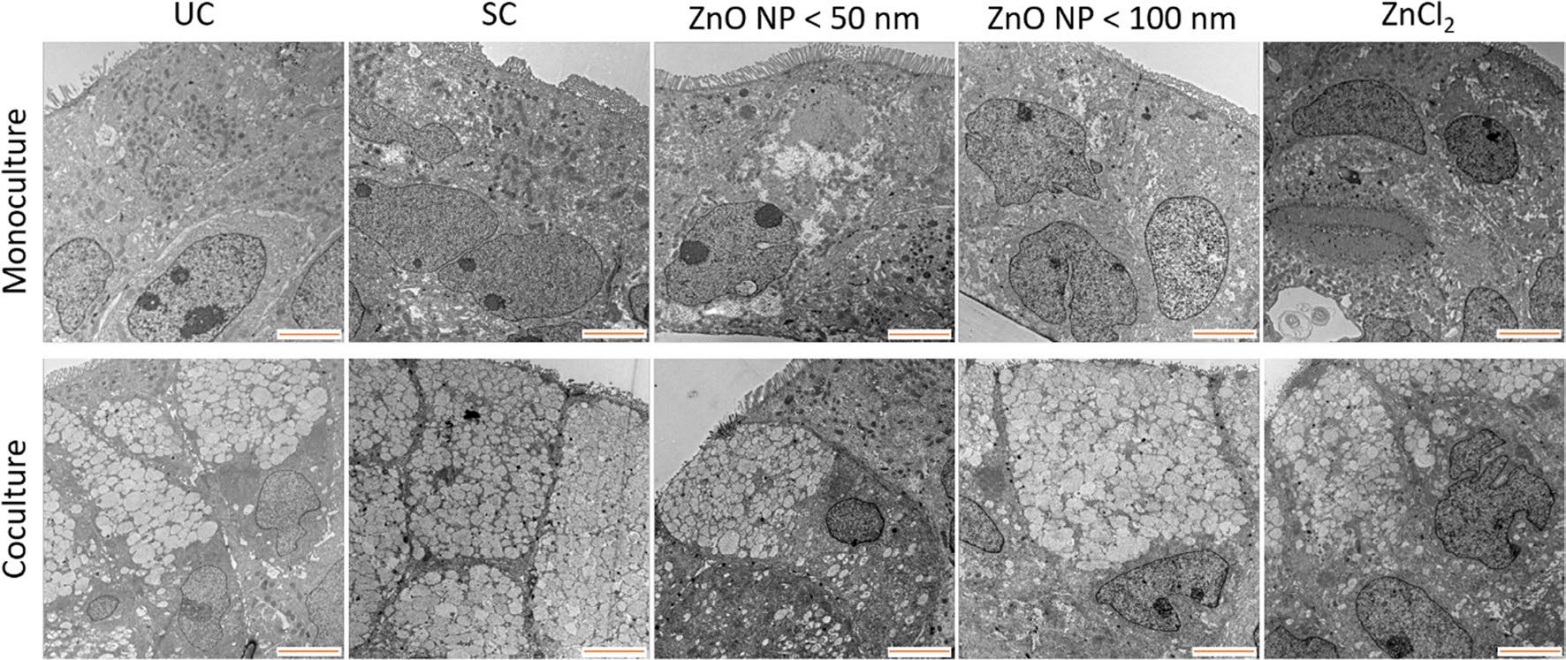
Representative TEM images of Caco-2 monocultured (upper row) and Caco-2/HT29-MTX cocultured cells (lower row) after 24 h of treatment with digested ZnO NP (< 50 nm and < 100 nm) and ZnCl_2_ (614 µM) as well as cell culture medium (untreated control = UC) and 10% intestinal juice (solvent control = SC). Orange scale bar: 5 μm

TEM was used to visualize organelles and cellular ultrastructure after treatment with digested ZnO NP (Fig. 5). Differentiated Caco-2 and HT29-MTX cells formed an epithelial polarized multilayer. Both cell types developed microvilli; there were clearly visible cell membranes, nuclei, and also cell-cell contacts. The cells treated with digested ZnO NP and ZnCl_2_ showed no abnormalities compared to the untreated cells and solvent control.

### Barrier integrity investigations

**Fig. 6:**
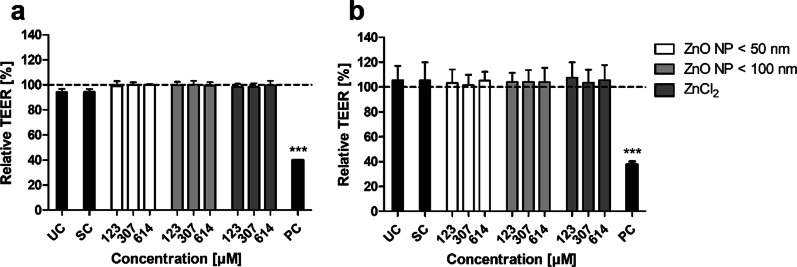
Relative TEER values of Caco-2 monocultured (**a**) and Caco-2/HT29-MTX cocultured cells (**b**) after 24 h of treatment with digested ZnO NP (< 50 nm and < 100 nm) and ZnCl_2_. UC: untreated control; SC: solvent control (10% intestinal juice); PC: positive control (0.1% triton X-100 + 10 mM EGTA apical, 10 mM EGTA basolateral). Dashed line (= 100%) implies TEER before incubation. Data are expressed as mean + SD; n = 3. Significant differences compared to UC (***P ≤ 0.001) were obtained by using the unpaired t test

The influence of digested ZnO NP on the barrier function of the monolayer was investigated by measuring TEER. Therefore, TEER values before and after 24 h of incubation were measured, and relative TEER values were calculated by normalizing the epithelial resistance before incubation to the epithelial resistance after incubation. Treatment of the Caco-2 monocultured cells (Fig. 6a) with the positive control triton X-100/EGTA resulted in significantly lower TEER values compared to the untreated control (94.2 ± 2.5% in the untreated control; 39.8 ± 0.4% in the positive control). Similar results were observed in the Caco-2/HT29-MTX coculture (Fig. 6b: 105.4 ± 11.6% in the untreated control; 37.4 ± 4.6% in the positive control). The intestinal juice solvent control as well as ZnO NP and ZnCl_2_ showed no influence on TEER with relative values higher than 94% in the monoculture and higher than 101% in the coculture.

**Fig. 7:**
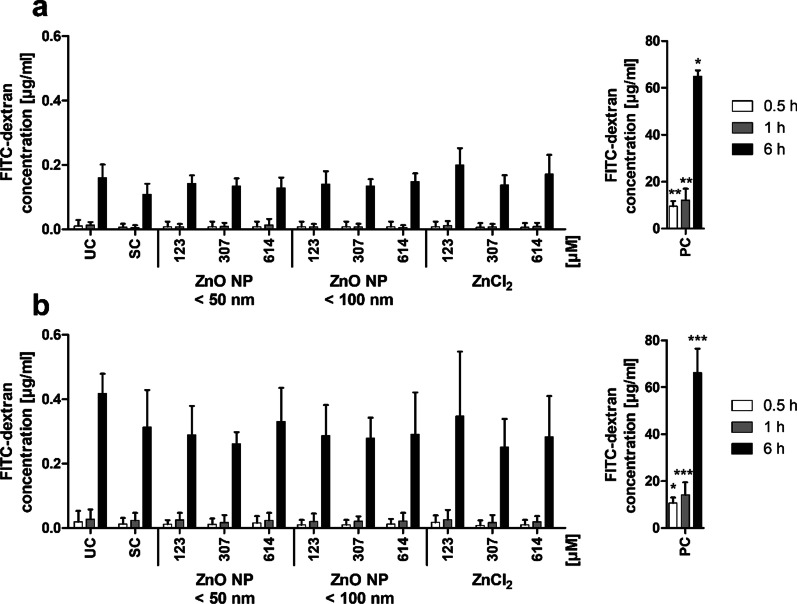
Basolateral FITC-dextran concentrations of Caco-2 monocultured (**a**) and Caco-2/HT29-MTX cocultured cells (**b**) after 24 h of treatment with digested ZnO NP (< 50 nm and < 100 nm) and ZnCl_2_. FITC-dextran was added in the apical compartment after cell incubation, and the basolateral was measured after 0.5 h (white bars), 1 h (grey bars), and 6 h (black bars). UC: untreated control; SC: solvent control (10% intestinal juice); PC: positive control (0.1% triton X-100 + 10 mM EGTA apical, 10 mM EGTA basolateral). Data are expressed as mean + SD; n = 4. Significant differences compared to UC (*P ≤ 0.05; **P ≤ 0.01; ***P ≤ 0.001) were obtained by using the unpaired t test

The influence of digested ZnO NP on the monolayer permeability was examined by measuring apically administered FITC-dextran in the basolateral compartment (Fig. 7). FITC-dextran passages membranes paracellularly. An increasing paracellular permeability leads to higher FITC-dextran amounts in the basolateral area. Our results showed, that there were higher FITC-dextran concentrations in the basolateral compartments in both cultures with increasing treatment time. Furthermore, the FITC-dextran concentrations were basically higher in the cocultured cells compared to the monoculture. The positive control triton X-100/EGTA led to significantly increased FITC-dextran concentrations in the basolateral compartments at all time points (monoculture: 9.5 ± 2.3 µg/ml after 0.5 h, 12.1 ± 4.9 µg/ml after 1 h, 65.0 ± 2.4 µg/ml after 6 h; coculture: 10.7 ± 2.3 µg/ml after 0.5 h, 14.1 ± 5.4 µg/ml after 1 h, 66.2 ± 10.3 µg/ml after 6 h) compared to the untreated control (monoculture: 0.01 ± 0.02 µg/ml after 0.5 h, 0.01 ± 0.01 µg/ml after 1 h, 0.16 ± 0.04 µg/ml after 6 h; coculture: 0.02 ± 0.03 µg/ml after 0.5 h, 0.03 ± 0.03 µg/ml after 1 h, 0.42 ± 0.06 µg/ml after 6 h). Treatment with the solvent control intestinal juice, ZnO NP, and ZnCl_2_ had no impact on the monolayer permeability.

**Fig. 8:**
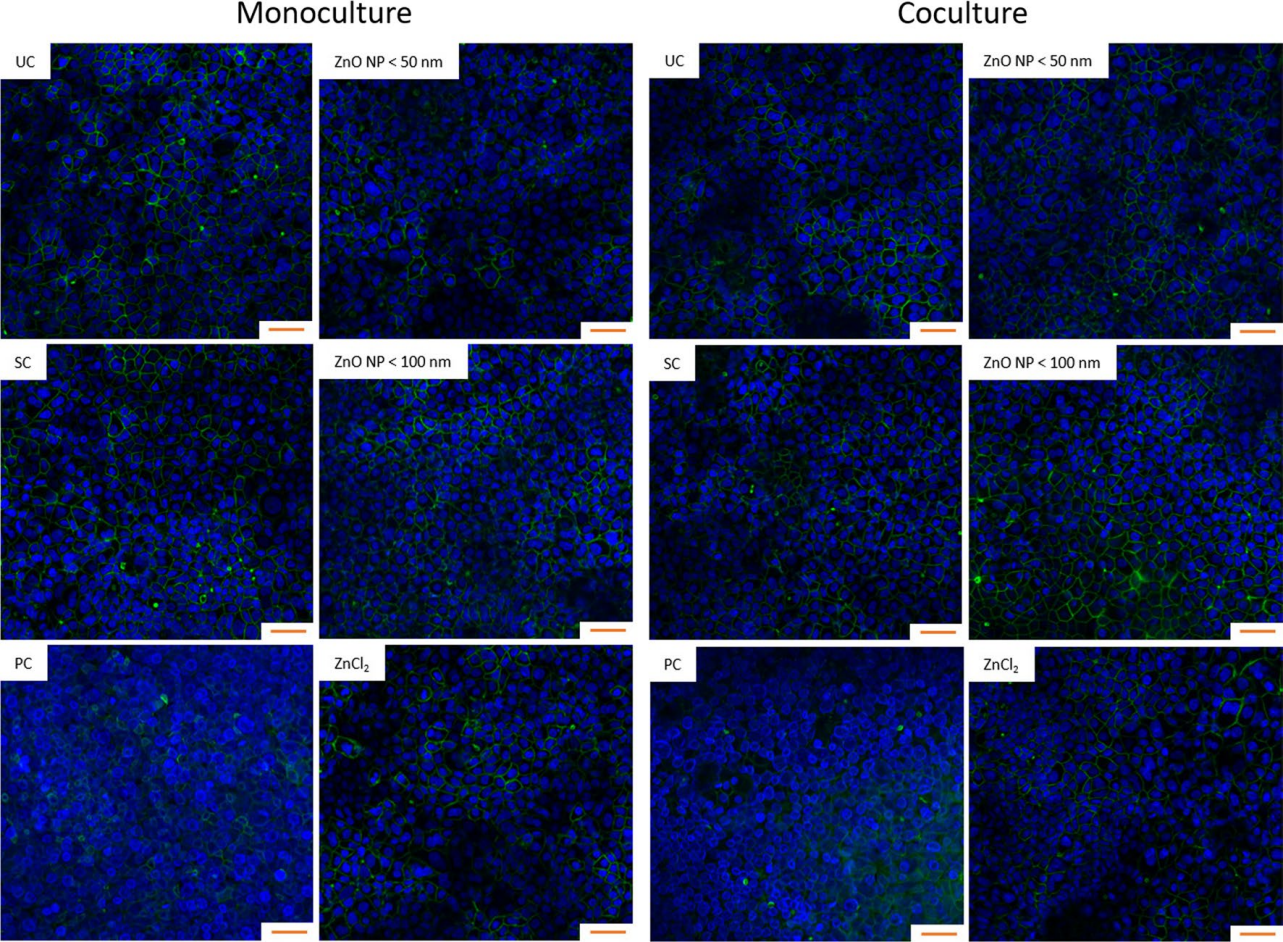
Fluorescence staining with phalloidin-iFluor 488 (green) and DAPI (blue) after 24 h of incubation with digested ZnO NP (< 50 nm and < 100 nm) and ZnCl_2_ (614 µM) of Caco-2 monocultured and Caco-2/HT29-MTX cocultured cells. UC: untreated control; SC: solvent control (10% intestinal juice); PC: positive control (0.1% triton X-100 + 10 mM EGTA apical, 10 mM EGTA basolateral); Orange scale bar: 50 μm

The impact of digested ZnO NP on the monolayer morphology was visualized via phalloidin-iFluor 488 and DAPI staining (Fig. 8). The monolayers of the Caco-2 monocultured and Caco-2/HT29-MTX cocultured cells showed similar morphological characteristics. The cells grew densely, and the nuclei and cytoskeletons were clearly visible. After treatment with the positive control triton X-100/EGTA, the cells appeared rounded and the monolayer was apparently detached from the Transwell insert. Incubation with the solvent control intestinal juice, ZnO NP, and ZnCl_2_ showed no influence on the cytoskeletal and nuclear morphology.

### Cytotoxicity assays

**Fig. 9:**
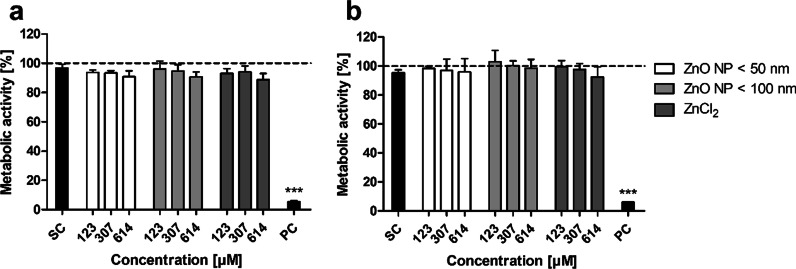
Relative metabolic activity of Caco-2 monocultured (**a**) and Caco-2/HT29-MTX cocultured cells (**b**) after 24 h of treatment with digested ZnO NP (< 50 nm and < 100 nm) and ZnCl_2_. SC: solvent control (10% intestinal juice); PC: positive control (0.1% triton X-100 + 10 mM EGTA apical, 10 mM EGTA basolateral). Data are normalized to the untreated control (= 100%; dashed line) and expressed as mean + SD; n = 3. Significant differences compared to the untreated control (***P ≤ 0.001) were obtained by using the unpaired t test

The metabolic activity of the Caco-2 monocultured and Caco-2/HT29-MTX cocultured cells was investigated using the MTT assay (Fig. 9). Data were normalized to the untreated control. Both mono- and cocultured cells showed a significantly decreased metabolic activity after treatment with the positive control triton X-100/EGTA (5.5 ± 0.6% residual metabolic activity in the monoculture; 5.9 ± 0.1% in the coculture). Treatment with the intestinal juice solvent control as well as ZnO NP and ZnCl_2_ resulted in no significant changes compared to the untreated control. The lowest metabolic activity was found after 24 h of incubation with 614 µM ZnCl_2_ (88.8 ± 4.3% in the monoculture; 92.3 ± 7.2% in the coculture).

**Fig. 10:**
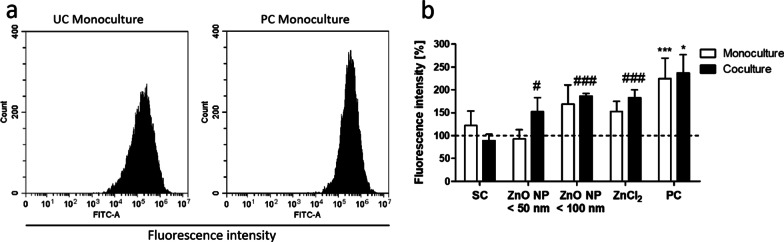
Intracellular detection of oxidative stress by flow cytometry using the DCFH-DA assay. **a** Histograms of the fluorescence intensities of the DCF dye in Caco-2 monocultured cells of the untreated control (UC) and the positive control (PC; 5 mM H_2_O_2_). The cells were treated with the probe DCFH-DA for 30 min. **b** Relative DCF fluorescence intensity in Caco-2 monocultured (white bars) and Caco-2/HT29-MTX cocultured cells (black bars) after 24 h of treatment with digested ZnO NP (< 50 nm and < 100 nm) and ZnCl_2_ (614 µM). SC: solvent control (10% intestinal juice). Data are normalized to the untreated control (= 100%; dashed line) and expressed as mean + SD; n = 3. Significant differences compared to the untreated control (*P ≤ 0.05; ***P ≤ 0.001) and to SC (^#^P ≤ 0.05; ^###^P ≤ 0.001) were obtained by using the unpaired t test

To investigate the ZnO NP-induced ROS in mono- and cocultured cells, the fluorescent redox active probe DCFH-DA was used (Fig. 10). Data were normalized to the untreated control. Treatment with the positive control hydrogen peroxide resulted in a more than twofold increase in the fluorescence intensity in both models compared to the untreated control (224.6 ± 37.0% in the monoculture; 237.2 ± 32.4% in the coculture). Incubation with the artificial intestinal juice resulted in a slightly increased fluorescence intensity in the monoculture (121.9 ± 25.4%) and a slight intensity decrease in the coculture (89.5 ± 11.0%). There were no significant changes in the DCF fluorescence intensity after treatment of the Caco-2 monocultured cells with both ZnO NP and ZnCl_2_ compared to the solvent control. In contrast, the comparable treatment of the Caco-2/HT29-MTX cocultured cells resulted in noticeable cellular ROS (152.6 ± 24.5% for ZnO NP < 50 nm; 186.6 ± 5.3% for ZnO NP < 100 nm; 183.3 ± 13.9% for ZnCl_2_) in comparison with the intestinal juice solvent control.

**Fig. 11:**
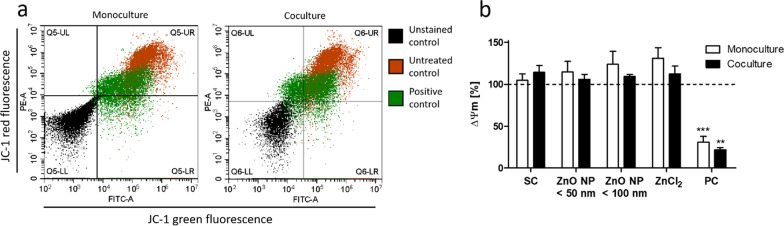
Mitochondrial membrane potential measured by flow cytometry using the JC-1 assay. **a** Dot plots of JC-1 aggregates (red fluorescence) and monomers (green fluorescence) in Caco-2 monocultured and Caco-2/HT29-MTX cocultured cells of the unstained, untreated, and positive control (PC; 100 µM CCCP). **b** Relative mitochondrial membrane potential (proportion of monomers and aggregates) of Caco-2 monocultured (white bars) and Caco-2/HT29-MTX cocultured cells (black bars) after 24 h of treatment with digested ZnO NP (< 50 nm and < 100 nm) and ZnCl_2_ (614 µM). SC: solvent control (10% intestinal juice). Data are normalized to the untreated control (= 100%; dashed line) and expressed as mean + SD; n = 3. Significant differences compared to the untreated control (**P ≤ 0.01; ***P ≤ 0.001) were obtained by using the unpaired t test

The effects of digested ZnO NP on the MMP in Caco-2 monocultured and Caco-2-/HT29-MTX cocultured cells were investigated by JC-1 staining (Fig. 11). Data were normalized to the untreated control. The positive control CCCP led to an impairment of the MMP compared to the untreated control (30.7 ± 7.2% in the monoculture; 21.7 ± 2.7% in the coculture). Treatment of both models with the artificial intestinal juice resulted in a similar red/green fluorescence intensity ratio compared to the untreated control, reflecting no changes in the MMP. Incubation with digested ZnO NP and ZnCl_2_ showed similar results as the intestinal juice. They neither increased nor decreased the MMP of the mono- and cocultured cells.

## Discussion

Because of their advantageous properties, ZnO NP are applied in a wide range of applications. The food sector is one of these important areas, with the result that ZnO NP can enter the human food chain [24]. The increasing oral exposure entails potential toxicity. In addition, the digestion process can change the physicochemical properties of ZnO NP, resulting in a different behaviour on cells or biomolecules [3]. However, so far, little is known about the fate of ZnO NP during digestion and about the effects of digested ZnO NP on the human gastrointestinal tract. Therefore, the aim of this study was to characterize two different sized ZnO NP during *in vitro* digestion and to investigate their effects on two intestinal model systems. For an approximation of physiological conditions, differentiated Caco-2 cells were used in monoculture and in coculture with mucus-producing HT29-MTX cells, which were grown in a Transwell system.

Characterization of the investigated ZnO NP is essential to evaluate their biological effects and for a better comparison with data from literature [27]. Both TEM and ICP-OES analyses showed that after *in vitro* digestion, most of the pristine ZnO NP changed their morphological structure and likely their chemical composition. About 70% of the zinc was available as free ions, and about 30% of the zinc was obviously bonded. There were only minor differences between ZnO NP and ZnCl_2_ regarding the proportions of free zinc ions and bonded zinc. Digested ZnO NP showed no structural similarities with the pristine large, rod-shaped particles, which we have already characterized [28].

A comparable biotransformation of ZnO NP after *in vitro* digestion was already seen in other studies. ZnO NP dissolved during the *in vitro* digestion process, and zinc ions were complexed with organic compounds from the enzymes and salts of the different digestive juices [3, 14]. In contrast to our investigations, ZnO NP completely dissolved in the gastric juice and there was a *de novo* particle formation with phosphate in the intestinal juice [3]. However, Yu and Choi [29] examined the *in vivo* solubility of different sized ZnO particles (< 100 nm and < 5 µM primary particle size) in rat gastric fluid. There was about 12% dissolved zinc for both particle types after oral administration. This indicates a low dissolution property of ZnO that is more influenced by the biological environment than the particle size, which is comparable to our results. Chia et al. showed that the dissolution behaviour of ZnO NP also depends on their coating properties [30]. The digestive process is complex with pH shifts and different enzymatic and ionic influences. This can even affect insoluble ZnO NP, which undergo physicochemical changes. It seems very likely that ZnO reaches the intestine not in the nanoparticulate form. Based on this, we have to accept that so far communicated effects of ZnO NP in cellular systems do not really or only partly reflect the consequences of an in vivo ZnO NP exposure.

In a first approach, the uptake as well as permeation of zinc through the monolayer were investigated after treatment of Caco-2 monocultured and Caco-2/HT29-MTX cocultured cells with digested ZnO NP. ICP-MS measurements revealed that the cellular amount of zinc increases with increasing ZnO NP concentrations. But, the zinc content in the basolateral compartments stayed low (0.1–0.4 µg/ml). TEM images revealed that cell components and organelles exhibited no abnormalities due to the increased cellular amount of zinc.

There were comparable results for ZnO NP and ZnCl_2_ treated mono- as well as cocultured cells. Li et al. [31] examined the bioavailability of digested ZnO NP for differentiated Caco-2 cells. They analysed the zinc content in the apical and basolateral compartments as well as the cellular zinc content. The bioavailability of zinc also increased with increasing applied ZnO NP quantities. In contrast to our investigations, their incubation medium contained lower zinc quantities (0.198–0.665 µM) than ours (123–614 µM). Nevertheless, they detected cellular changes after treatment with digested ZnO NP. Caco-2 cells exhibited less microvilli and more vacuoles in the mitochondria. The cells were dehydrated but contained more fat particles, and the size of the endoplasmic reticulum increased. Interestingly, there was no solvent control to exclude the effects caused by the intestinal juice [31]. Similar morphological changes after treatment of intestinal cells with digested ZnO NP were detected in the study of Moreno-Olivas et al. [14]. There was a disruption of the brush border membrane of differentiated Caco-2/HT29-MTX cells after 4 h of treatment with digested ZnO NP (~ 1190 µM). Lower concentrations of ZnO NP (0.12 µM and 11.9 µM) showed no influence on the monolayer morphology. For this electron microscopic analysis, the cells were grown for only two weeks in 6-well plates with no division in an apical and basolateral compartment. It could already be shown that a differentiation period of less than 21 days leads to a lower generation of transporter systems and protein expression, and the monolayer is less confluent and differentiated [32]. Nevertheless, zinc is an essential trace element, which is primarily absorbed by enterocytes in the small intestine [33]. Therefore, an increased zinc uptake by Caco-2 monocultured and Caco-2/HT29-MTX cocultured cells is a logical consequence.

The low amounts of zinc in the basolateral compartments indicated an intact barrier function of the monolayer, which was investigated afterwards. There were no changes in TEER, permeability, and cytoskeletal and nuclear morphology after 24 h incubation of Caco-2 monocultured and Caco-2/HT29-MTX cocultured cells with digested ZnO NP and ZnCl_2_.

Moreno-Olivas et al. [14] found increased TEER values of Caco-2/HT29-MTX cocultured cells after 4 h of treatment with digested ZnO NP (1190 µM) compared to the solvent control, which was substantiated with strengthened tight junctions due to the zinc exposure. Lower concentrations of ZnO NP (0.12 µM and 11.9 µM) showed TEER values similar to the solvent control. It is already known that zinc supplementation can improve the intestinal epithelial barrier function [34]. Interestingly, the permeability was basically higher in Caco-2/HT29-MTX cocultured cells than in Caco-2 monocultured cells. This may be reasoned by the fact that tight junctions are not that firm in HT29-MTX coculture models [35]. They express less tight junctions compared to Caco-2 cells, and the paracellular permeability of hydrophilic compounds is higher in coculture [36, 37]. However, treatment of both mono- and cocultured cells with digested ZnO NP did not lead to an increased permeability compared with the solvent control.

Although there were no cellular morphological changes, zinc uptake may have resulted in cytotoxic damage, which was also investigated in our study. The metabolic activity and mitochondrial membrane potential of Caco-2 monocultured and Caco-2/HT29-MTX cocultured cells were not influenced by the treatment with digested ZnO NP and ZnCl_2_. However, increased intracellular oxidative stress led to a significantly increased DCF fluorescence in the coculture after 24 h of incubation with both ZnO NP and ZnCl_2_.

Li et al. [31] detected cytotoxic effects of digested ZnO NP on differentiated Caco-2 cells using the MTT and lactate dehydrogenase assay. There were dose-dependent effects with up to 50% reduced metabolic activity and a threefold higher lactate dehydrogenase release after treatment with digested ZnO NP (0.198–0.665 µM zinc content in the digestate). The ROS levels increased with concentration [31]. Notably, there were no positive and digestate controls in all assays, which impedes the validity of the results. In the study of Moreno-Olivas et al. [14], differentiated Caco-2/HT29-MTX cocultured cells were stained with calcein AM/propidium iodide for live/dead staining after 4 h of treatment with digested ZnO NP. There were no changes in the number of dead cells, but a significant increase in the number of live cells was observed for the highest ZnO NP dose (1190 µM). The authors found no logical explanation for this. They only concluded that cell death is more likely to be caused by digestion components than NP [14].

The generation of ROS is often postulated as the origin of ZnO NP induced toxicity [7, 38]. This involves, for instance, increased amounts of malondialdehyde, nitrite and hydrogen peroxide/hydroxyl radicals, and reduced glutathione, catalase, and superoxide dismutase content [11, 39, 40]. In general, zinc has antioxidant effects and zinc deficiency is linked with oxidative stress [41, 42]. However, the influence of zinc within the cellular oxidant/antioxidant balance is complex [42]. In in vitro studies, treatment of cells with ZnO NP led to a disruption of cellular zinc homeostasis, resulting in the generation of ROS and oxidative stress [43].

It has already been shown, that undigested ZnO NP can alter the MMP of various cell types [11, 43–46]. But to our knowledge, this is the first study, which investigated the impact of digested ZnO NP on the MMP of intestinal cells. Altogether, the unmodified MMP of Caco-2 monocultured and Caco-2/HT29-MTX cocultured cells after treatment with digested ZnO NP maintains the absent toxicity of digested ZnO NP in physiological relevant concentrations.

During *in vitro* digestion, chemical, biological, and physical conditions change depending on pH shifts and the attendance of different enzymes and salts. ZnO NP undergo transformation with altered physicochemical properties and zinc ion release, which leads to a modified bioavailability and toxicological behaviour against organisms [27]. Zinc ions can be bound by ligands, but zinc ions binding is reversible and dynamic [14]. There are several in vivo animal studies, that have investigated the toxicity of ZnO NP after oral exposure. In a study by Liu et al. [23], mice were fed with 2000 mg ZnO NP per kg for 270 days. The intestinal ZnO NP uptake was only marginal; consequently, there was no accumulation in tissues and organs. ZnO NP yielded similar results as bigger ZnO particles and zinc ions, which is comparable with our results. Other studies also verified the absence of animal toxicity after oral exposure of physiological ZnO NP concentrations [15, 21, 22, 47].

To further improve our used in vitro intestinal models, the cocultured intestinal cells could be complemented with immune cells, such as THP-1, thus reaching an even better approximation to the *in vivo* situation. In this context, further especially immunological endpoints, for example the release of cytokines or antioxidant markers, should be included.

## Conclusions

The *in vitro* digestion of ZnO NP leads to a physicochemical transformation, which was expressed by morphological changes from mouth to intestinal phase, and a high zinc solubility. Treatment of mono- and cocultured cells with digested ZnO NP results in a dose-dependent zinc uptake. But the internalized zinc has no impact on the cell morphology or organelles, and the metabolic activity and MMP are not influenced. TEER, permeability, and the morphology of the cytoskeleton and nuclei are not changed. Overall, the in vitro digested ZnO NP have no toxic effects at concentrations of 123–614 µM on differentiated Caco-2 monocultured and Caco-2/HT29-MTX cocultured cells and their intact monolayer barriers prevent zinc from reaching the basolateral area in an uncontrolled manner. It seems very likely that zinc reaches the intestine not in the nanoparticulate form and possibly binds to other components, which should be considered by investigations of effects after oral uptake of ZnO NP.

## Materials and methods

### Materials

Alpha-amylase, CaCl_2_ ∙ 2H_2_O, 4′,6′-diamidino-2-phenylindole (DAPI), 2′,7′-dichlorofluorescin-diacetate (DCFH-DA), MgCl_2_ ∙ 6H_2_O, mucin, ox bile, pancreatin, paraformaldehyde, pepsin, trypsin and ZnO nanopowder (#677450 and #544906) were purchased from Sigma-Aldrich Chemie GmbH, Taufkirchen, Germany. Dulbecco’s Modified Eagle Medium (DMEM), foetal bovine serum (FBS), non-essential amino acids, penicillin/streptomycin, and trypsin/EDTA were obtained from PAN-Biotech GmbH, Aidenbach, Germany. 3-(4,5-Dimethylthiazol-2-yl)-2,5-diphenyl tetrazolium bromide (MTT), dimethyl sulfoxide, fluorescein isothiocyanate (FITC)-dextran, hydrogen peroxide (30%), nitric acid (Suprapur), and triton X-100 were acquired from Merck KGaA, Darmstadt, Germany. 2-((3-Chlorophenyl) hydrazinylidene) propanedinitrile (CCCP) and ZnCl_2_ were procured from Thermo Fisher Scientific Inc., Waltham, MA, USA. Carbamide, ethylene glycol tetraacetic acid (EGTA), KCl, KH_2_PO_4_, NaCl, NaHCO_3_, and Na_2_HPO_4_ ∙ 2H_2_O were purchased from Carl Roth GmbH & Co. KG, Karlsruhe, Germany. Cacodylic acid and glutaraldehyde were bought from Serva Electrophoresis GmbH, Heidelberg, Germany. 5,5′,6,6′-Tetrachloro-1,1′,3,3′-tetraethylbenzimidazolylcarbocyanine iodide (JC-1) was obtained from Enzo Life Sciences GmbH, Lörrach, Germany. Phalloidin-iFlour 488 reagent was acquired from Abcam plc., Cambridge, UK.

### Preparation of ZnO NP dispersions and in vitro digestion procedure

ZnO nanopowder was dispersed in 8.5 g/l NaCl (ZnO target concentration 3.34 mg/ml). The dispersions were filled in a glass rosette cell (RZ2; Bandelin electronic GmbH & Co. KG, Berlin, Germany), which was submerged in an ice bath to avoid heat production. An ultrasonic homogenizer (Bandelin Sonopuls HD 2070) was plunged 1 cm deep in the dispersions. The NP were ultrasonicated with a critical sonication energy of 720 J/ml for 1 h to break NP agglomerates and aggregates. The in vitro digestion was conducted after DIN 19738 [48] and Stein et al. [49]. The sonicated dispersions were further diluted with synthetic saliva (8.5 g/l NaCl, 41.55 U α-amylase) to obtain 15 ml of three different ZnO NP concentrations (3.34, 1.67, and 0.67 mg/ml). Skimmed milk powder was additionally added and served as the food matrix. To mimic the mouth phase of digestion, all samples were placed in a shaking water bath (GFL Gesellschaft für Labortechnik GmbH, Burgwedel, Germany) for 5 min at 37 °C and 120 rpm. Afterwards, the gastric phase was simulated by adding 35 ml of gastric juice (0.7 g/l KCl, 0.27 g/l KH_2_PO_4_, 2.9 g/l NaCl, 3 g/l mucin, 1 g/l pepsin, pH 2). The samples were placed again in a shaking water bath for 2 h at 37 °C and 120 rpm. Subsequently, the intestinal phase was imitated by the addition of 50 ml intestinal juice (0.5 g/l CaCl_2_ ∙ 2H_2_O, 0.3 g/l KCl, 0.2 g/l MgCl_2_ ∙ 6H_2_O, 1 g/l NaHCO_3_, 9 g/l ox bile, 0.3 g/l carbamide, 9 g/l pancreatin, 0.3 g/l trypsin, pH 7.5). All samples were positioned in the shaking water bath for 3 h at 37 °C and 120 rpm. For the cell culture experiments, the digested dispersions were finally mixed 1:10 with the cell culture medium to obtain concentrations of 10, 25, and 50 µg/ml (equivalent to 123, 307, and 614 µM).

### Characterization of digested ZnO NP dispersions

Inductively coupled plasma optical emission spectrometry (ICP-OES) was used to determine the portion of free zinc ions and bound zinc in the intestinal juice. For this purpose, the digested dispersions were centrifuged for 60 min at 5250×*g* (Allegra X-15R; Beckman Coulter GmbH, Krefeld, Germany). Supernatants were transferred into new containers. Precipitates were dried for 6 h at 95 °C until mass constancy. Supernatants were further diluted and acidified at a ratio of 1:10 with extra pure nitric acid. Precipitates were suspended in 1 ml 65% extra pure nitric acid and 0.5 ml hydrogen peroxide and placed in a sonication bath (Transsonic 460/H; Elma Schmidbauer GmbH, Singen, Germany) for 30 min at 50 °C. The precipitates were filled up with ultrapure water to 50 ml. A dilute solution for yttrium was added to all samples as an internal standard element. The total zinc content in the supernatants and precipitates was measured by ICP-OES (iCAP™ 7000 equipped with an CETAC ASX-520 autosampler, both from Thermo Fisher Scientific Inc., Waltham, MA, USA) using two different wavelengths, 206.2 and 334.502 nm. Two quality-check samples (QC; certified reference material) within the concentration range of the analytical results were chosen and measured regularly after about 20 samples. The minimum number as well as the acceptance criteria for evaluating the QC are in accordance with the Food and Drug Administration guidelines [50]. The limit of quantification was calculated in compliance with DIN 32645 [51] as 0.3 ± 0.1 mg/l. The measurement of uncertainty was determined with two certified reference materials according to DIN ISO 11352:2013-03 [52] to be 2.3%. Data processing and quality management were conducted with an in-house developed program (from Chemical Analytics, German Environment Agency, Bad Elster, Germany) and Qtegra™ (Thermo Fisher Scientific Inc., Waltham, MA, USA).

Furthermore, the fate of ZnO NP during *in vitro* digestion was investigated using transmission electron microscopy (TEM). After each digestion step, samples were taken and 4 µl of each sample was placed on a Formvar-carbon filmed 400 mesh grid (Quantifoil Micro Tools GmbH, Großlöbichau, Germany). The samples were dried and analysed using a Zeiss CEM 902 A electron microscope (Carl Zeiss AG, Oberkochen, Germany). Images were acquired with a wide-angle dual-speed 2 K-CCD-camera (TRS, Moorenweis, Germany).

### Cell culture and ZnO NP treatment

Mono- and coculture conditions were used to mimic the human intestine, considering that enterocytes and goblet cells represent the two major intestinal cell types [53]. The human epithelial cell line Caco-2 originates from a colon carcinoma and is typically used as a model of the intestinal barrier. Caco-2 cells exhibit the ability to differentiate, and so, they receive morphological and functional similarities to absorptive enterocytes in the small intestine. They develop a characteristic polarization with a brush border containing enterocyte-specific enzymes and cell-cell contacts [54]. Additionally, the human colon adenocarcinoma cell line HT29 was used. HT29 cells show characteristics of mature intestinal cells and are, therefore, used for investigations of food digestion and bioavailability. They can also differentiate to acquire other morphological and functional properties. The addition of methotrexate (MTX) into the cell culture medium induces the differentiation to HT29-MTX cells. They are similar to goblet cells of the small intestine and are able to produce mucus [55].

Caco-2 and HT29-MTX cells were cultured in DMEM supplemented with 10% FBS, 1% non-essential amino acids, and 1% penicillin/streptomycin in an incubator (37 °C, 95% humidity, 5% CO_2_; Thermo Fisher Scientific Inc., USA). At regular intervals, the cells were tested for mycoplasma contamination. Both cell lines were verified by short tandem repeat profiling. For all assays, cells were seeded on ThinCert™ cell culture inserts in 12-well plates (3 μm pore size; Greiner Bio-One International GmbH, Germany). Caco-2 cells were used in mono- and also in coculture with HT29-MTX cells (ratio 3:1; altogether 3.3 × 10^5^ cells per well). The cells were cultivated for 23 days to receive a differentiated and stable mono- and coculture. The cell culture medium was changed every 2–3 days. Differentiated cells were treated with 123–614 µM digested ZnO NP dispersed in cell culture medium. The choice of the concentrations was based on the study by Sohal et al. [24]. They calculated physiologically relevant *in vitro* doses depending on the daily human intake of NP. There is no information about the daily ZnO NP intake. However, TiO_2_ is applied in comparable areas, especially in the food sector. Therefore, it is assumed that TiO_2_ and ZnO NP are taken up in similar quantities (5.4 mg/kg bodyweight/day). This results in an estimated *in vitro* dose of about 246 µM [24]. Zinc chloride served as a source of free zinc ions and was applied in equimolar concentrations as ZnO. Cell culture medium was used for the untreated control. The solvent control consisted of intestinal juice diluted with cell culture medium (1:10) to exclude possible effects caused by the solvent. As positive control, 0.1% triton X-100 and 10 mM EGTA on the apical side and 10 mM EGTA on the basolateral side diluted with cell culture medium were used.

### Quantification of zinc

Inductively coupled plasma mass spectrometry (ICP-MS) was used to investigate the uptake and permeation of digested ZnO NP through the monolayer. For the examination of the cellular amount of zinc, the cells were harvested after 24 h of exposure to ZnO NP. The cells were washed with phosphate-buffered saline (PBS), and Trypsin/EDTA was added for 5 min at 37 °C. Afterwards, DMEM with 10% FBS was added and the Transwell inserts were rinsed until the cells detached. The detached cells were centrifuged (5 min at 600xg; centrifuge 5810R; Eppendorf AG, Hamburg, Germany). The supernatants were removed, and the cells were washed with PBS. Cell viability and number were determined using the CASY TT (OLS OMNI Life Science GmbH & Co. KG, Bremen, Germany), and the cells were centrifuged again (5 min at 600×*g*). The cell pellets were diluted with 65% extra pure nitric acid and digested over-night. To support the cellular digestion, the samples were placed in a sonication bath (Transsonic 310/H; Elma Schmidbauer GmbH, Singen, Germany) for 1 h at 50 °C. Subsequently, the samples were diluted with purified water to 2% nitric acid. The ionic zinc content was measured using the ICP-MS iCAP™ RQ (Thermo Fisher Scientific Inc., Waltham, MA, USA) equipped with the 4DX prepFAST autosampler (ESI Elemental Service & Instruments GmbH, Mainz, Germany). The mass signal m/z = 66, which corresponds to the zinc isotope with a mass of 66 amu, was determined. Rhodium was used as an internal standard with a concentration of 2 ppb in 2% nitric acid. It was continuously added to the samples and quantified simultaneously. Three QC were chosen within the measurement range and measured regularly after about 20 samples. The minimum number and acceptance criteria for evaluating the QC were in agreement with the FDA guidelines [50]. The limit of quantification was calculated after DIN 32645 [51] as 0.3 ± 0.3 µg/l, and the measurement uncertainty was calculated according to DIN ISO 11352:2013-03 [52] with three certified reference materials as 6.9%. Data processing was performed with an in-house developed program (from Chemical Analytics, German Environment Agency, Bad Elster, Germany) for quality management as well as Qtegra™ (Thermo Fisher Scientific Inc., Waltham, MA, USA).

In addition, zinc permeation through the monolayer was determined, which allows indirect conclusions about the cellular zinc uptake. The incubation dispersions were measured before treatment of the mono- and cocultured cells and afterwards from the apical and basolateral compartments. All samples were acidified with extra pure nitric acid, and the total ionic zinc content was quantified under the same conditions as described above. The limit of quantification was determined as 1.4 ± 0.7 µg/l.

### Transmission electron microscopy evaluation

TEM was used to investigate the possible cellular consequences that result from the uptake of digested ZnO NP. For this, the cells were treated with 614 µM ZnO NP and ZnCl_2_ for 24 h. Afterwards, the cells were washed twice with PBS to remove NP residues from the cellular surface. For a primary fixation, 2.5% glutaraldehyde was added for 1 h at room temperature. The cells were washed two times with cacodylic acid sodium salt buffer (100 mM, pH 7.2) for 15 min. Finally, the Transwell insert membranes were excised and further processed as already described [56].

### Barrier integrity investigations

Different approaches were used to investigate the influence of ZnO NP on the monolayer barrier integrity. By measuring the transepithelial electrical resistance (TEER), conclusions can be drawn about the monolayer integrity and the integrity of tight junctions as well as the ionic conductance of the paracellular pathway in the epithelial monolayer [57]. The ohmic resistance was measured both before and after 24 h of incubation with ZnO NP to obtain relative TEER values using chopstick electrodes and an epithelial Volt/Ohm meter (EVOM2; World Precision Instruments GmbH, Friedberg, Germany).

Furthermore, FITC-dextran was used to investigate changes in the monolayer permeability due to the treatment with ZnO NP. Dextran will be transported transcellularly through the monolayer depending on its permeability. Dextran can then be quantified photometrically because of the linked FITC marker. After 24 h exposure to ZnO NP, the incubation medium was replaced by phenol red-free DMEM on the apical and basolateral compartments for 10 min to wash the cells. DMEM was removed again, and 500 µl 1 mg/ml FITC-dextran was added on the apical side and 1000 µl phenol red-free DMEM was added on the basolateral compartment. The cells were incubated at 37 °C on an orbital shaker for 6 h. After 30 min, 60 min, and 6 h, the basolateral DMEM was pipetted in triplicate onto a 96-well plate. The fluorescence was measured with a microplate reader (excitation: 485 nm, emission: 528 nm; Synergy 2; BioTek Instruments, Inc., Bad Friedrichshall, Germany), and the FITC-dextran concentration was calculated using a standard series (0.001–1000 µg/ml).

To examine morphological changes after ZnO NP treatment, the cells were stained with phalloidin-iFluor 488 and DAPI to visualize actin filaments in the cytoskeleton and cell nuclei. For this purpose, mono- and cocultured cells were treated with digested ZnO NP and ZnCl_2_ for 24 h. After two wash steps with PBS, they were fixed with 4% paraformaldehyde for 20 min. They were washed again and permeabilized with 0.1% triton-X 100 for 10 min. The cells were washed once more and stained with 0.1 µg/ml DAPI and 0.1% phalloidin-iFluor 488 for 1 h. To analyze the samples, the Transwell insert membranes were excised and transferred onto a microscopic slide. The evaluation was carried out using a laser scanning microscope (excitation: 488 nm, emission: 445 nm for DAPI and 525 nm for phalloidin-iFluor 488; LSM 780; Carl Zeiss AG).

### Cytotoxicity assays

The potential cytotoxic impact of digested ZnO NP was assessed by conducting different investigations. To determine the cellular metabolic activity, the MTT assay as an accepted and common cytotoxicity test was used [58]. After 24 h of treatment with ZnO NP, the cell culture medium was removed and 0.5 mg/ml MTT was added to the apical and basolateral compartments (stock solution: 50 mg MTT powder in 10 ml PBS; working solution diluted with DMEM). The cells were incubated for 2 h at 37 °C. MTT was removed, dimethyl sulfoxide (≥ 99.8%) was added to the apical and basolateral compartments to solubilize the resulting formazan, and the plate was placed on an orbital shaker for 10 min. Finally, apical and basolateral dimethyl sulfoxide was mixed, and the samples were placed in triplicate onto a transparent 96-well plate. The absorbance was measured at 570 nm using a microplate reader (reference wavelength: 630 nm; Synergy 2; BioTek Instruments, Inc., Germany).

To detect ROS, the DCFH-DA assay was used. After 24 h incubation with digested ZnO NP, the cells were harvested as described above. The cells were centrifuged (5 min at 600×*g*), and the cell pellet was mixed with 1 µM DCFH-DA. The cells were incubated for 30 min at 37 °C. Hydrogen peroxide (5 mM, 30 min) served as a positive control after the DCFH-DA treatment. A CytoFLEX flow cytometer (Beckman Coulter GmbH, Krefeld, Germany) was used to measure 1 × 10^4^ viable cells per sample (excitation: 488 nm, emission: 525 nm). Data processing was carried out using the CytExpert software (Beckman Coulter GmbH, Krefeld, Germany).

The membrane-permeant JC-1 dye was used to investigate the MMP. After 24 h of exposure to digested ZnO NP, the cells were harvested as described above. The cells were centrifuged (5 min at 600×*g*) and the supernatants were removed. The positive control was incubated with 100 µM CCCP for 5 min. All samples were treated with 8 µM JC-1 for 15 min at 37 °C. After washing the cells with PBS, 1 × 10^4^ viable cells per sample were measured using the CytoFLEX flow cytometer (Beckman Coulter GmbH, Krefeld, Germany). The excitation wavelength was 488 nm. The emission was measured using two different wavelengths to differentiate between JC-1 monomers (525 nm) and aggregates (585 nm). The MMP results from the proportion of monomers and aggregates, which was evaluated using the CytExpert software (Beckman Coulter GmbH, Krefeld, Germany).

### Statistical analysis

At least three independent experiments were performed for each assay. IBM SPSS Statistics (Version 26; IBM Corporation, Armonk, NY, USA) was used for data processing. To detect significant differences, one-way analysis of variance with the Ryan–Einot–Gabriel–Welsh post hoc test or the unpaired t test were conducted. The figures were generated using GraphPad Prism 5 (GraphPad Software, San Diego, CA, USA). The results shown in the figures represent means and standard deviations.

## Data Availability

The datasets used and/or analysed during the current study are available from the corresponding author on reasonable request.
